# Meeting of Delegates

**Published:** 1884-07

**Authors:** B. H. Catching

**Affiliations:** Atlanta, Ga.


Atlanta, Ga., June 14, 1884.

### Meeting of Delegates.

Delegates to the National Dental Association, and others who may act in a representative capacity, will meet in Washington City, July 22, at the Ebbitt House, where reduced rates have been made. Reduced railroad rates may be obtained by unity of action on the part of those who will attend from States or sections.

Everything indicates a large meeting for organization. Enough

States have already elected delegates to insure a good attendance and a profitable meeting.

The acceptability of the *plan* has been shown by the large number of favorable responses from every section of the United States.

The profession of America will unite for one purpose in a common cause, to wit: To build up the name and honor of our calling. An organization will be put forth in which self will be lost to the good of the *order*. Unity of action will be brought about, brotherly love from every section will be promoted, and an association formed that will fill the heart of every American dentist with pride, and stimulate to higher aims and nobler purposes. B. H. CATCHING, Correspondent.

